# Tulsa Oklahoma Oktoberfest Tent Collapse Report

**DOI:** 10.1155/2012/729795

**Published:** 2012-05-10

**Authors:** Kelly E. Deal, Carolyn K. Synovitz, Jeffrey M. Goodloe, Brandi King, Charles E. Stewart

**Affiliations:** ^1^OTEMS (Oologah-Talala EMS), 18955 S 4150 Road, Claremore, OK 74017-3609, USA; ^2^Department of Emergency Medicine, University of Oklahoma School of Medicine, Tulsa, OK 74135, USA; ^3^Oklahoma Disaster Institute, Tulsa, OK 74135, USA

## Abstract

*Background*. On October 17, 2007, a severe weather event collapsed two large tents and several smaller tents causing 23 injuries requiring evacuation to emergency departments in Tulsa, OK. *Methods*. This paper is a retrospective analysis of the regional health system's response to this event. Data from the Tulsa Fire Department, The Emergency Medical Services Authority (EMSA), receiving hospitals and coordinating services were reviewed and analyzed. EMS patient care reports were reviewed and analyzed using triage designators assigned in the field, injury severity scores, and critical mortality. *Results*. EMT's and paramedics from Tulsa Fire Department and EMSA provided care at the scene under unified incident command. Of the 23 patients transported by EMS, four were hospitalized, one with critical spinal injury and one with critical head injury. One patient is still in ongoing rehabilitation. *Discussion*. Analysis of the 2007 Tulsa Oktoberfest mass casualty incident revealed rapid police/fire/EMS response despite challenges of operations at dark under severe weather conditions and the need to treat a significant number of injured victims. There were no fatalities. Of the patients transported by EMS, a minority sustained critical injuries, with most sustaining injuries amenable to discharge after emergency department care.

## 1. Introduction

On October 17, 2007 a severe weather alert was issued for Tulsa, Oklahoma, and the surrounding area by the Tulsa office of the National Weather Service of the National Oceanic and Atmospheric Administration (NOAA). This weather alert forecast two separate fronts moving through the area separated by about 2 hours. Each of these fronts was forecast to produce thunderstorms and high winds (Appendix D). 

At the time, Tulsa was hosting an annual outdoor Oktoberfest celebration, considered to be the third largest in the world. Although the venue had not yet opened to the general public, corporate sponsors, event organizers, and volunteers were in attendance for “corporate night.” More than 7,000 people were present at the festival when the second storm front arrived, spawning tornadoes and winds up to 80 miles per hour. These winds collapsed two large tents and several smaller ones at approximately 1930 hours. Twenty-three people were transported by EMS to area hospital emergency departments, with six listed in serious condition and two listed as critical. The others were reported in fair condition. In addition to these patients transported by EMS, others injured at the event were either evaluated and released on scene or sought medical care after self-extrication and leaving via personal transportation.

### 1.1. Location: Tulsa

Situated on the Arkansas River at the foothills of the Ozark Mountains in northeast Oklahoma, a region of the state known as “Green Country,” Tulsa is the second-largest city in the state of Oklahoma and 45th-largest in the United States. With an estimated population of 382,872 and approximately 950,000 in the statistical metropolitan area according to the 2006 census, Tulsa serves as the governmental seat of Tulsa County, the most densely populated county in Oklahoma. Located near “Tornado Alley”, the city frequently experiences severe weather.

### 1.2. Event: Oktoberfest

The Tulsa Oktoberfest is operated by Oktoberfest, Inc. (a nonprofit organization). The profits from the Oktoberfest are used in Tulsa's River Parks to create new playgrounds for the city's children and to make other improvements to the parks. The event site is located on the west bank of the Arkansas River, across the river from all major hospitals in Tulsa. Although the festival is located across the river from the major hospitals, there are no access problems to these hospitals by established routes.

## 2. Methods

Retrospective data analysis and interviews of investigator-selected responders at the scene were approved by the University of Oklahoma Health Science Center Institutional Review Board (IRB). Interviews were conducted using a script approved by the IRB (Attachment 1).

EMS patient assessment and care data were obtained from computerized EMS transportation records. Protected data was carefully redacted per NSA Report number I333-015R-2005 guidelines.

Injury severity scores were calculated by the senior author (C. E. Stewart) and reviewed by two other authors (J. M. Goodloe and C. K. Sinuitz). One author was the EMSA MMRS Director who responded to the incident (K. E. Deal), and one author was on duty at the hospital that received the bulk of both walk-in and transported patients (J. M. Goodloe).

## 3. Results

The following is a brief description of the events that occurred at the Oktoberfest site as reported by the various agencies, hospitals, and individuals that were involved.

### 3.1. Emergency Medical System Response

The Medical Emergency Response Center (MERC) was notified by NWS-Tulsa in a conference call of the impending weather at about 1400 hours. This briefing included the chance of high winds and severe thunderstorms. NWS forecasters felt that if a warming trend occurred, these storms could spawn tornadoes. NWS also mobilized the local SkyWarn amateur radio network of storm spotters. (Appendix A is the 1111 AM updated weather forecast.)

Following these specific and accurate warnings given by NWS, the Metropolitan Medical Response Service (MMRS) Director at MERC initiated a severe weather protocol that mobilized all EMS supervisors and administrators, readied all available ambulances for deployment (including those with drivable conditions that were in maintenance garages), and prestocked resupply vehicles with additional equipment, cervical collars, and trauma supplies. The MMRS Director cancelled an impending personal leave and staffed his response vehicle with an EMT who was also an amateur radio operator. These two personnel monitored the SkyWarn amateur radio frequency for severe weather and tornado warnings.

The first weather front passed at approximately 1600 hours with only minor damages including a motor vehicle accident on US Route 169. Following the passage of the storm front, clear skies prevailed. The local warming trend that NWS had predicted would worsen the oncoming second front that began to occur.

NWS issued a high wind/severe storm warning at 1858 hours for Tulsa County. A tornado watch was already in effect for Tulsa County at that time. The second storm front passed through the southern Tulsa area at approximately 1920 hours, just as darkness fell. This storm was accompanied by winds in excess of 70 mph (on-site estimates ranged as high as 80 mph). SkyWarn volunteers noted cyclonic rotation of the storm, though no tornadoes were noted in the Tulsa area.

Public warnings were limited to radio, television, and NOAA broadcast storm warnings. Severe storm warning sirens were not activated based on the city of Tulsa policy limiting wind-based use of weather sirens to winds of 80 miles per hour or greater. As the storm front hit, Oktoberfest staff felt that the safest area was under the tents due to rain and approximately 1/2 inch sized hail accompanying the storm.

It is unknown whether the Oktoberfest staff had knowledge of the second NWS severe storm warning prior to the arrival of the storm front. Many of the sponsors were local television stations and their weather offices did call their staff on site with the threat. One television station did advise their staff to try and shut down the event due to the predicted severity of the storm's second front.

The second storm front picked up the leading (southwestern) edge of Der Bier Garten tent (labeled number 6 in the Oktoberfest event map) with about 2500 people under the canvas. Wind pressure raised the tent lifting the canvas like a parachute. The corner posts held firm, so the interior 400 pound tent poles were lifted off of the ground. This tent pole destabilization occurred three times, with injuries occurring each time. During the third wind gust, one tent pole was entrapped by falling tent fabric and vertical motion was converted to a horizontal scything motion. The sweeping tent pole struck multiple victims and indiscriminately flung equipment and tables about. Collapse of the Der Bier Garten tent occurred 24 minutes after the gust front arrived. Simultaneously, a smaller tent, number 3 on the diagram, Die Bierstube, also collapsed. Heavy rain accompanied the high winds and the sun had set, so the area was in darkness.

At approximately 19 : 27 on October 17, 2007, the 911 dispatcher received approximately 30 phone calls within a 10-minute period. The first EMS vehicle on scene was dispatched for a single patient, and the reporting caller gave no mention of multiple patients. Although multiple calls arrived in rapid succession, it was not until the fourth or fifth phone call that the tent collapse was reported. Subsequent calls detailed a collapse of tents at the Oktoberfest and noted that multiple injuries had occurred. The dispatcher immediately vectored multiple ambulances to the area and notified fire and police to respond. The first responding EMS unit arrived within 2 minutes of the first call.

The first responding ambulance was met by several hundred people who directed the ambulance in conflicting directions. The ambulance proceeded into the crowd and was unable to provide effective care or transportation due to the crowd. Multiple responders described dozens of individuals with bleeding head and extremity wounds, walking to cars or aiding other victims.

The first responding supervisor (and EMS unit) was the EMSA MMRS Director, who responded to the series of 911 calls. He established an incident command site, directed incoming ambulances to a staging area, and established liaison between police and EMS. The responding supervisor intercepted subsequent units and issued ingress instructions. When the fire department arrived, a unified incident command was established.

As noted earlier, the first arriving unit was unable to egress the area due to the crowd. Accordingly, the first incident command direction was for the police to establish open egress and ingress to the area. This was done within 5 minutes.

Tulsa Fire Department responded with five units including Rescue 4, Ladder 4, Engine 26, Ladder 26, and District Chief car 641. This response included 15 personnel. The responding District Chief assumed command of the fire department personnel at the scene.

The MERC Coordinator started hospital notification of the disaster and updated the hospitals at via EMResource (a proprietary real-time MCI event notification and hospital capability status website). There were six subsequent MCI event status updates sent via EMResource to Tulsa area hospitals during the progression of the mass casualty event.

Initial victim identification and triage proved challenging aside from the environmental milieu. EMTs and paramedics from the Tulsa Fire Department were directed to multiple clusters of reported victims, often by confusing and contradictory bystander directions. There were at least 3 areas where bystanders were simultaneously trying to rally EMS presence.

The MMRS Director was in constant contact with the Tulsa Area Emergency Management Agency (TAEMA) to assure continued weather monitoring during the incident response. A weather satellite review revealed no further incoming hazardous weather. Relatively early into the event, he was advised by TAEMA that there was no additional weather threat expected.

A central triage verification and treatment area was established in an unaffected tent which was carefully evaluated for structural integrity, arriving EMSA paramedics gathered in this central area to receive subsequent assignments.

**Figure 1:**
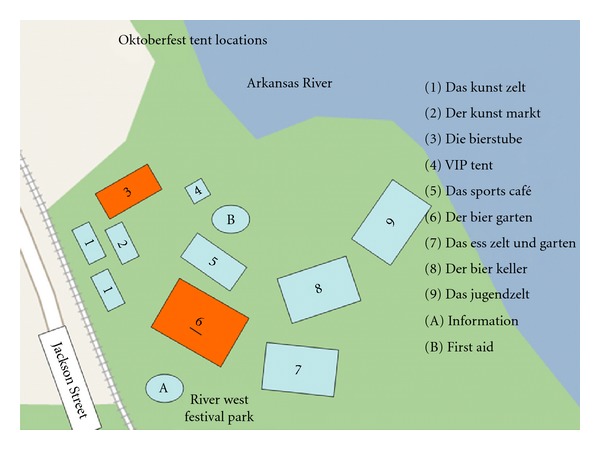


A map of the Oktoberfest event obtained by an on-site internet query proved to be inaccurate, reflecting last year's Oktoberfest tent configuration (Figure 1). After noting that the event maps were not accurate, incident command established “left,” “right,” and “central” casualty areas in relation to the treatment and transport sites. These tactical designators proved helpful in resolving confusing terminology for the victim locations. EMSA EMTs remained in the ambulances to ensure timely mobilization of these vehicles for patient transport and ongoing ingress/egress clearance. Subsequent arriving field supervisory EMSA paramedics sequentially staffed these positioned ambulances.

All EMS-transported patients were triaged and tagged accordingly. These triaged patients were moved to the central treatment area by Tulsa Fire personnel. For unexplained reasons, triage tags were removed from three patients at the juncture of extrication and treatment. Re-identification of these patients rapidly occurred without significant clinical impact upon patient outcome. A total of 23 patients were ultimately transported by 9 EMSA ambulances. Several ambulances were able to make multiple transports due to the proximity of area hospitals to the MCI event site.

### 3.2. Casualty Transport Destination Distribution

**Figure 2:**
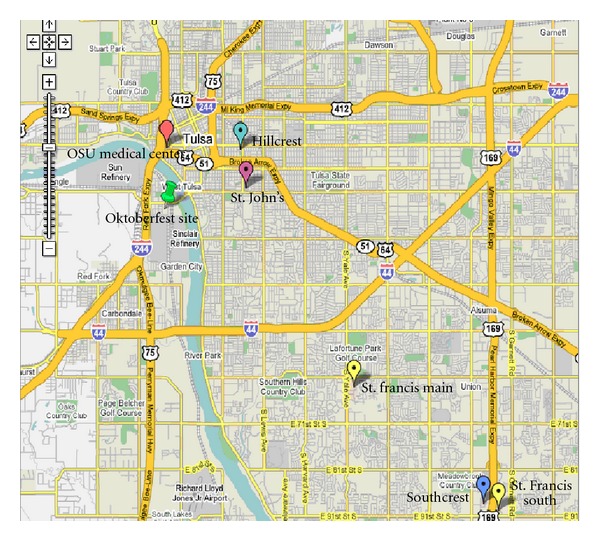
Legend: red = OSU Medical Center, light blue = Hillcrest Medical Center, maroon = St. John's Medical Center, yellow = St. Francis Hospital (Main) on Yale Avenue, yellow = St. Francis Hospital (South) at bottom of map, dark blue = Southcrest Hospital, Green “tack” = site of Oktoberfest celebration.

Review of casualty transport destinations reveals that EMS-transported patients were equitably transferred and distributed throughout the Tulsa acute care hospitals (Figure 2). Distribution of the patients by MERC in coordination with incident command and transport/triage at the scene was in a “far first” pattern based on the Israeli model with transportation of the first patients to the furthest hospitals equipped to receive casualties [1]. Patients were sent into hospitals in a “trauma rotation,” to ensure reasonably equitable distribution of transported casualties.

One of the authors (J. M. Goodloe) was the attending emergency physician at Saint Francis Hospital during this MCI. Personal observation of this emergency physician indicated minimal impact on typical emergency department operations at this hospital. Ancillary and nursing staff attended to the received casualties without duress.

Four “walking wounded” patients were transported by a “lift” bus with three paramedics in attendance to the furthest hospital from the scene (St. Francis South). (These transports and their calculated injury severity scores are described in Table 1.)

One critical patient was transported with spinal injuries and subsequent paraplegia. One seriously ill patient was transported with concussion, loss of consciousness, and head injury.

A total of 35 walking wounded casualties presented to local hospitals later that evening. The bulk of these presented to the largest hospital in Tulsa (Saint Francis Hospital) and to SouthCrest Hospital which was further away from the Oktoberfest event than Saint Francis Hospital. (The available information about these patients is detailed in Table 2).

It was assumed that the walking wounded casualties would present to the closest hospital (Oklahoma State University Medical Center). This assumption was found to be in error with this population. The reasons for this distribution cannot be conclusively determined but are felt to be demographically related to the site of the personal residences of the participants. (On this evening only the vendors and sponsors were celebrating and the majority of these would live in the southern part of the city due to socioeconomic characteristics.)

## 4. Discussion

Since the events of September 11, 2001, and the more recent Hurricane Katrina in 2005, significant attention has been focused on mass casualty preparedness and response. In Oklahoma, severe weather is common with tornadoes and severe thunderstorms occurring frequently during about one-fourth of the year.

Convectively generated windstorms occur over broad temporal and spatial scales as was seen in this event. The longer-lived, larger-scale, and most intense of these windstorms are given the name “derecho.” Individual derechos have been responsible for up to 8 fatalities, 204 injuries, and forest blowdowns affecting over 3,000 km^2^ of timber [3]. These losses totaled $500 million. When casualty statistics and damage estimates from hurricanes and tornadoes are contrasted with those from derechos, it is obvious that derechos can be as hazardous as tornadoes and hurricanes [3]. This windstorm was part of a derecho that affected the plains states and extended into the Ozarks.

A derecho is associated with a fast-moving band of severe thunderstorms. Derechos are usually not associated with a cold front, but a stationary front within a highly buoyant, warm air mass. A warm weather phenomenon, derechos occur mostly in summer, especially July (in the northern hemisphere), but can occur at any time of the year and occur as frequently at night as in the daylight hours. They occur commonly only in North America.

Derecho comes from a Spanish word for “straight”. The word was first applied to the storm front in the American Meteorological Journal in 1888 by Gustavus Hinrichs [4]. He intended to contrast this with tornado, which comes from the Spanish word “tornar” meaning “to turn”. Derechos come from a band of thunderstorms that are bow- or spearhead-shaped and hence are also called a bow echo or spearhead radar echo.

Multiple instances of tent collapse with fatalities and severe injuries have been reported in the media as a result of windstorm, but similar instances have been reported only twice in the medical literature available for online searching [5–15]. Tent collapse due to high wind is well known to the owners of marquee tents. EMS and fire have little documentation of this as a hazard of severe weather; thus, public safety preparedness efforts do not commonly include this hazard.

The details presented and described Previously comprise the City of Tulsa's emergency services response to the uncommon consequence of a common natural hazard in Oklahoma. EMSA had nine ambulances staffed by 21 paramedics and EMTs and seven supervisors who coordinated triage, transport, and logistics with the Tulsa Fire Department and Tulsa Police Department.

Although this was a structural collapse, the response scene at the time of emergency services personnel arrival was structurally safe. All power lines were rendered safe by the Oktoberfest management within moments of the incident, so Tulsa Fire Department actions were allocated completely to patient care without the competing needs of fire suppression, structural stabilization, or hazard mitigation.

### 4.1. Role of the Media

The media plays a vital role during disasters as the chief source of public information [16]. Mass communication is also critical to public safety to ensure that appropriate information is passed to the public and panic is prevented. The media has to be monitored and handled with care, so information is delivered as precisely as possible during a disaster. This event was no exception.

During the Oktoberfest tent collapse, the police department staged the media across the street from the tent collapse and the triage areas. This location gave them event coverage, preserved patient confidentiality, and ensured that media did not negatively impact clinical operations during the event. The police department managed the media until the Public Information Officer (PIO) for EMSA arrived, approximately 50 minutes after the initial 911 calls. There was some difficulty with her gaining access to the scene that further delayed EMSA management of the media response.

Local television, radio, and newspaper agencies were present on site. Information was given by telephone to some state and national media outlets, though most used local affiliate reports. Briefings occurred and individual interviews were granted on site through 2200 hours; telephone and e-mail updates were provided through the overnight hours and the following day. Local reports conveyed key messages (such as, “Citizens should not report to the site”) and positively portrayed rescue efforts for the two-hour event. The six local hospitals experienced no problems with the media at their facilities. A press briefing was given the day following the tent collapse, and all hospitals, EMSA, Tulsa Fire Department, and other responders were represented in this briefing.

### 4.2. After-Action Report

Several important points were made during after-action discussion. Multiple agency-specific debriefings occurred. A multiagency debriefing was co-coordinated by the Tulsa Area Emergency Management Agency and was well attended by all participants. There was widespread acknowledgement that tent collapse should be a factored hazard of severe weather. Specific to this event, the following items were identified for further hazard planning education and operations.

1. In this crowded nighttime venue, it was difficult for EMS and fire providers to locate the command post and patient collection area.
2. NWS weather warnings should have been distributed to the event planners.
  - With the abundant warning of the oncoming storm, evacuation of the event would have been relatively easily accomplished in a timely fashion.
  - This would have prevented all injuries to the crowd.
3. A map of the event area and location of tents should have been provided to EMS, fire, and police providers so that orientation of incoming units could be planned and coordinated.
  - Although the map in Figure 2 was available online, there were no copies distributed to police, fire, or EMS. Indeed, responding supervisors found a different placement of tents than was depicted on the map which ultimately proved representative of the 2006 Oktoberfest.
  - Emergency access and egress lanes should be planned/provided for fire, police, and EMS vehicles.
4. Further training about triage and triage tag use for multiple casualty responses was requested.
5. EMS/fire supervisors should be integrated in weather warnings and have access to weather channels.
6. The city of Tulsa is considering “special event” planning for large attendance community events. Specific requirements would include hazard(s) identification and emergency service command post, patient treatment and transport sectors, and staging point location determinations as well as ingress and egress routes.

## Figures and Tables

**Table 1:** Transported Patient Summary. Injury severity code = sum of squares of the AIS grade in each of the three most severely injured areas as described by Baker et al. [2]. Injury severity score for these transported patients was assigned from description in the transport records, vital signs, and was verified by three of the authors.

Facility	Age	Sex	Reported Injury	Transport Mode	ISS
OSU Medical Center	36	M	Head and jaw trauma (Immobilized)	Ambulance	9
	50	M	Laceration to finger	Ambulance	4
	48	M	Head trauma—no spinal immobilization	Ambulance	4

Hillcrest Medical Center	23	F	Back pain (immobilized)	Ambulance	9
	25	M	Head trauma (immobilized)	Ambulance	9
	55	M	Leg pain	Ambulance	4
	24	F	Closed head injury	Ambulance	9

St. Francis Hospital	40	M	Head and neck pain—LOC	Ambulance	16 + 4 = 20
	39	M	Head injury (immobilized)	Ambulance	9
	33	F	Shoulder trauma. Head injury—immobilized	Ambulance	4 + 4
	32	F	Hit on head by tent pole (immobilized)	Ambulance	9

Southcrest Hospital	29	M	Head laceration	Ambulance	9
	42	F	Hip pain, right ankle pain, left elbow pain	Ambulance	4 + 4 + 1
	26	F	Back pain	Ambulance	9

Saint Francis South Hospital	32	M	Facial and eye injuries	Transit bus with paramedic	4
	57	M	Leg injury (abrasion)	Transit bus with paramedic	1
	1	F	Scalp injury	Transit bus with paramedic	4
	44	M	Leg injury	Transit bus with paramedic	1

St. John Medical Center	54	F	Back pain	“Walking wounded” Ambulance EVAC	4
	48	F	Back pain (immobilized)	Ambulance	9
	34	M	Head laceration (immobilized)	Ambulance	9
	54	M	Head trauma (immobilized)	Ambulance	9
	46	F	Spinal Trauma (immobilized—paralyzed)	Ambulance	25

Totals	A total of 23 patients were identified as transported to a Tulsa emergency department from the Oktoberfest tent collapse.				

**Table 2:** 

Facility	Age	Gender	Reported Injury	Outcome
OSU Medical Center	48	M	Back and head injury	Treated/Released
	37	M	Back and neck pain	Treated/Released
	57	M	Finger injury	Treated/Released

Hillcrest Medical Center	??	??	Head laceration	Treated/Released

St. Francis Hospital	23	M	??—Oktoberfest mentioned in patient history	Treated/Released
	33	F	??—Oktoberfest mentioned in patient history	Treated/Released
	41	M	??—Oktoberfest mentioned in patient history	Treated/Released
	25	F	Hit on head by tent pole	Treated/Released
	38	M	Hit on head by tent pole	Treated/Released
	29	M	Hit on head by tent pole	Treated/Released
	53	F	??—Oktoberfest mentioned in patient history	Treated/Released

Southcrest Hospital	Southcrest Hospital stated that they had 9 presenting patients but would not provide any further information.			

Saint Francis South Hospital	45	M	Leg injury	Treated/Released

St. John Medical Center	St. John Medical Center stated that they had 4 presenting patients but would not provide any further information.			

Totals	A total of 25 patients were identified as self-presenting to a Tulsa emergency department and related to the Oktoberfest tent collapse.			

## Appendices

### A. Interview Script

*Tulsa MMRS Oktoberfest Injury Analysis Interview Script*

All interviewees must be informed before interview.

1. All answers are voluntary. You may decline to participate. There is no requirement to complete the questions once started. If you wish that the information given not included in the final paper, you may request that at any time before the research is published.
2. Please make responses general to protect the anonymity of responders and civilians.
3. This is a confidential interview.
4. No personal information will be recorded.

These questions concern the October 17, 2007, Medical Response at the Oktoberfest in Tulsa, Oklahoma.

1. What was your general role during the response?
2. How were you notified of the incident?
3. What do you think were the biggest strengths of the response?
4. What are you most impressed by in this response?
5. What is your opinion of the coordination of agencies during the response?
  - How could this be improved?
6. What was the most hazardous problem you encountered in providing patient care?
  - How did you deal with it?
7. How do you think this incident could have been mitigated (lessened) or prevented?
8. What were the biggest problems that you encountered during the incident?
9. Do you have any ideas that might improve a medical response to such an incident in the future?
10. How did your previous training or experience prepare you for this incident?
11. What actions would you change that happened during this incident?
12. What actions really worked during this incident?
13. How can we improve next time this happens?
14. Do you have a story that helps illustrate this event?

### B. The Weather Report

NATIONAL WEATHER SERVICE TULSA OK

1111 AM CDT WED OCT 17 2007

.UPDATE…..

SIGNIFICANT SEVERE WEATHER EVENT EXPECTED LATE THIS AFTERNOON AND

EVENING WITH THE POTENTIAL FOR STRONG/LONG LIVED TORNADOES…

LINE OF THUNDERSTORMS DEVELOPED IN RESPONSE TO A STRONG LOW LEVEL

JET OVER THE CENTRAL PART OF OKLAHOMA…AND WAS MOVING TO THE

NORTH NORTHEAST THIS MORNING…WITH STRONG TO SEVERE THUNDERSTORMS

OCCURRING WITHIN THIS LINE. THE MAJORITY OF THIS ACTIVITY WILL

AFFECT NORTHEAST OKLAHOMA INTO THE EARLY AFTERNOON…WITH STRONG

WIND AND MARGINALLY SEVERE HAIL POSSIBLE. THIS LINE WILL LEAVE A

TEMP/DEWPOINT GRADIENT ACROSS NORTHEAST OKLAHOMA…WHICH MAY

PROVIDE A FOCUS FOR THE LOCATION/MOVEMENT OF STORMS THAT DEVELOP

ALONG THE DRY LINE THIS AFTERNOON. THE TEMP GRADIENT MAY BE

FURTHER ENHANCED NOW THAT THE CLOUDS ARE BEGINNING TO THIN ACROSS

EASTERN OKLAHOMA AND NORTHWEST ARKANSAS.

### C. Typical Severe Weather Clause in Tent Rental Agreement

*Weather*

Client understands that tents are temporary structures designed to provide limited protection from weather conditions, primarily sun and rain; however there may be situations, particularly those involving strong winds and lightning, in which the tents will not provide protection and may even be damaged or blown over. Evacuation of tents to avoid possible injury is recommended when severe weather threatens the area where the tents are erected. People must leave the tents and not seek shelter in tents during such conditions. It is best to evacuate when in doubt. Marquee Tent offers an on-sight technician during the event for an additional charge to assist with weather assessment and equipment maintenance. If client declines those services, client understands that it is client's responsibility to be aware of changing weather conditions and to exercise its best judgment with regard to the evacuation of tents. client agrees that in the event of a predicted or actual storm or excessive winds, Marquee Tents may dismantle any equipment that has been previously installed to ensure safety of all involved.

### D. Critical Information Dissemination—Oktoberfest Event, October 17, 2007, National Weather Service, Tulsa

The wind damage that occurred across Tulsa County, including Oktoberfest, on the evening of October 17, 2007, was the result of a thunderstorm downburst. The downburst produced wind gusts of up to around 85 mph across Tulsa County, which resulted in fairly widespread straight-line wind damage. The peak measured wind gust during the event was observed at the Tulsa International Airport at around 727 pm, when an instrument measured an 85 mph gust. Wind gusts that were experienced at Oktoberfest were likely no more than 85 mph.

As noted in the product issuance timeline hereinafter, the National Weather Service issued a Tornado Watch that included Tulsa County at 226 pm, a Severe Thunderstorm Warning for Tulsa County at 702 pm, and a follow-up Severe Weather Statement to update the warning for Tulsa County at 714 pm. Estimated time of the Oktoberfest damage was at 723 pm.

The following information was disseminated from the NWS Office in Tulsa on October 17, 2007 preceding the damage in Tulsa County, which included the Oktoberfest Event. NWS Tulsa actually began highlighting the possibility of a significant severe weather event in its products as early as Friday, October 12. The following items *summarize *what was issued from this office in the preceding 18 hours.

151 am: Area Forecast Discussion highlighting “significant severe weather event late this afternoon and evening”.

335 am: Zone Forecast Product “occasional showers and thunderstorms today—some thunderstorms may be severe this afternoon. Rain chance 90 percent. Showers and thunderstorms likely in the evening—some thunderstorms may be severe. Rain chance 60 percent.”

345 am: Public Information Statement “review of severe weather safety rules” prior to “an outbreak of severe thunderstorms across eastern Oklahoma and northwest Arkansas”, including tornado, lightning, hail, and damaging straight-line wind.

500 am: Hazardous Weather Outlook “severe weather outbreak expected by late afternoon and evening with the possibility of strong tornadoes…large hail and damaging straight-line winds”.

743 am: Tornado Watch 708 until 4 pin including Tulsa County and surrounding counties.

924 am: Area Weather Summary “severe weather anticipated”.

1028 am: Zone Forecast Product updated to increase evening rain chance to 70 percent.

1111 am: Area Forecast Discussion “significant severe weather event expected late this afternoon and evening”.

1153 am: Hazardous Weather Outlook “significant risk of tornadoes, hail to baseball size and wind gusts to 75 mph”.

1200 pm: GoToWebinar briefing for Emergency Managers and state officials; TAEMA attended the briefing.

1245 pm: telephone briefing for TAEMA, which included timing of severe weather into Tulsa County that evening, possibility of tornadoes, hail to tennis ball size, and wind gusts in excess of 80 mph with severe storms.

1248 pm: Area Forecast Discussion “a second round of strong to severe thunderstorms could move into the forecast area by 1930z”.

144 pm: Significant Weather Alert through 230 including Tulsa for small hail and 50 mph wind gusts

221 pm: Area Forecast Discussion (mesoscale) increasing severe weather threat this afternoon; increasing tornado potential this evening.

222 pm: Significant Weather Alert through 3 pm including western Tulsa County for small hail and 50 mph wind gusts.

226 pm: Tornado Watch 711 through 10 pm including western Tulsa County and surrounding counties.

230 pm: Zone Forecast Product updated to include new tornado watch and to increase rain chance tonight to 80 percent.

241 pm: Area Forecast Discussion (mesoscale) severe potential increasing with ongoing storms in eastern Oklahoma and round 3 developing on dry line to the west.

245 pm: Significant Weather Alert through 330 pm including Tulsa County for small hail and 50 mph wind gusts.

257 pm: Area Forecast Discussion “parameters continue to align for a significant severe weather outbreak this evening”.

331 pm: Significant Weather Alert through 415 pm including Tulsa County for small hail and wind gusts to 50 mph.

346 pm: Severe Thunderstorm Warning through 445 pm for Tulsa County (penny hail and wind gusts to 60 mph).

354 pm: Severe Weather Statement updating warning (penny hail and wind gusts to 60 mph).

401 pm: Area Forecast Discussion (mesoscale) numerous reports of downed trees in Tulsa and wind gust of 62 mph in west Tulsa; thunderstorms in central OK will move into the area this evening and significant wind damage…very large hail…and tornadoes are likely.

413 pm: Severe Weather Statement cancelling SVR for Tulsa County.

415 pm: Local Storm Report noting several high wind and hail reports for Tulsa County.

633 pm: Severe Thunderstorm Warning though 745 pm for Creek County (penny size hail and 60 mph wind gusts).

634 pm: Short Term Forecast through 745 pm “strong to severe thunderstorms will be affecting portions of” Osage… Pawnee.. Washington… Creek… Okfuskee…Tulsa…and Nowata Counties.

639 pm: Area Forecast Discussion (mesoscale) increasing severe weather threat with storms moving northeast from Lincoln County.

702 pm: Severe Thunderstorm Warning through 815 for Tulsa County (penny size hail and 60 mph wind gusts).

708 pm: Hazardous Weather Outlook addressing tornado potential, golf ball size hail and wind gusts to 80 mph (potential).

713 pm: Severe Weather Statement updating Creek County warning (penny size hail and wind gusts to 70 mph).

714 pm: Severe Weather Statement updating Tulsa County warning (wind gusts to 70 mph).

727 pm: Local Storm Report mentioning thunderstorm wind damage at Oktoberfest at 723 pm with injuries; penny hail report 3 miles east of Sapulpa at 719 pm.

731 pm: Severe Weather Statement canceling Creek County warning.

728 pm: Local Storm Report mentioning thunderstom1 wind gust of 63 mph at Tulsa International Airport.

737 pm: Severe Weather Statement updating Tulsa County warning (wind gusts to 70 mph).

8 11 pm: Severe Weather Statement expiring Tulsa County warning.
